# Functional and Radiological Outcomes of Miniature Plate Osteosynthesis in Metacarpal and Phalangeal Fractures of Hand: A Prospective Study

**DOI:** 10.7759/cureus.58759

**Published:** 2024-04-22

**Authors:** Raj Singh, Pankaj Kumar Sharma, Vinit Verma, Vinod Kamboj, Ajay Shoeran, Jyoti Sharma

**Affiliations:** 1 Orthopedics and Trauma, Pandit Bhagwat Dayal Sharma Post Graduate Institute of Medical Sciences, Rohtak, IND; 2 Orthopedics, All India Institute of Medical Sciences, Bathinda, Bathinda, IND; 3 Orthopedics and Trauma, Holy Heart Multispecialty Hospital, Rohtak, IND; 4 Orthopedics and Traumatology, General Hospital Ambala, Ambala, IND; 5 Orthopedics, Pandit Bhagwat Dayal Sharma Post Graduate Institute of Medical Sciences, Rohtak, IND; 6 Anesthesiology, All India Institute of Medical Sciences, Bathinda, Bathinda, IND

**Keywords:** skilled worker, functional outcome, unstable fractures, phalangeal fracture, metacarpal fracture, miniature plate osteosynthesis

## Abstract

Introduction: There are several operative modes to address hand fractures to gain better anatomical and functional results including external fixator, percutaneous K-wire fixation, lag screw fixation, tension band wiring, intra-medullary nails or wires, and plate-screw fixation. We evaluated the results of plate osteosynthesis in fractures of metacarpals and phalanges in a prospective manner.

Material and methods: A total 50 adults (19-60 years) of either sex having 58 fractures managed by miniature plate osteosynthesis and followed for a minimum six months (6-19), including metacarpal and phalangeal fractures (unstable or serial fractures), intra-articular fractures, fracture-dislocation of proximal interphalangeal and distal interphalangeal joints with joint incongruity or subluxation were enrolled while contaminated compound fractures, pathological fractures and cases of reimplantation were excluded from study. Clinical assessment was done using the American Society for Surgery of the Hand (ASSH), total active flexion (TAF), total active range of motion (TAM) score, and the Disabilities of the Arm, Shoulder, and Hand (quick DASH) score while sequential radiographs were done at each follow-up.

Result: All the fractures had perfect union clinically as well as radiologically with a mean duration of six weeks while functional outcomes in reference to clinical scores were observed excellent and fair in all cases. None of the cases had any loss of reduction, loosening of the implant, or other major complications.

Conclusions: Miniature plate fixation provides enough stability in metacarpal and phalanges fractures, thereby allowing immediate active movements, which leads to excellent functional outcomes and early return to normal activities.

## Introduction

Fractures of hand bones are not uncommon and account for major injured parts of the body, constituting 10% of all fractures [1]. Border metacarpals (MC) are injured mostly and are more commonly at the base of the 1st and neck of the 5th MC. Hand fractures may be presented either by deformity (no treatment) or stiffness (overtreatment) and with both deformity and stiffness from poor treatment [1,2]. Most of the fractures in hands can be easily treated with conservative methods including splinting or plaster of Paris immobilization in the functional position of the limb [3,4]. There are several operative modes to gain better anatomical and functional results including external fixator, percutaneous k-wire fixation, lag screw fixation, tension band wiring, intra-medullary nails or wires, and plate-screw fixation [4-8]. It is a better approach to address these injuries while treating surgeons who are familiar with all the modes of treatment, in order to tailor a specific technique for a particular injury and patient [5]. There is no clear consensus regarding the ideal method of treatment with diverse modalities and their results. Meanwhile, a smaller number of prospective studies have been documented on the treatment of unstable MCs and phalangeal fractures using miniature plates and screws with excellent functional outcomes [8-11]. We evaluated prospectively the functional and radiological outcomes of miniature plate osteosynthesis in fractures of MCs and phalanges.

## Materials and methods

This prospective study was approved by the institutional ethical board with study approval number, IRB/Dean/16/1640-45 (Institutional Ethical Review Board, Pandit Bhagwat Dayal Sharma Post Graduate Institute of Medical Sciences, Rohtak). The written and informed consent were taken from all the patients. The study enrolled 50 patients (58 fractures) of either sex having MC or proximal phalangeal fractures presented in the orthopedic emergency department at the author’s tertiary care center. These were managed by open reduction and internal fixation (ORIF) with miniature plate osteosynthesis and followed for at least six months over seven years of duration (April 2013 to March 2020). Fracture patterns included unstable and serial MC/phalange fractures, intra-articular fractures, and fracture-dislocation of proximal interphalangeal (PIP) joints (with joint incongruity or subluxation), while Gustilo and Anderson grade II and III (IIIa, b, and c) compound fractures, pathological fractures and cases of reimplantation were excluded from the study. Patients who also had severe polytrauma, neurological and head injuries, and systemic musculoskeletal ailments hampering the rehabilitation of hands were also excluded.

Surgical technique

All the procedures were performed with all aseptic precautions in the supine position using suitable anesthesia and under pneumatic tourniquet control. For MC fractures, a dorsal longitudinal incision was made with a curve at the distal or proximal ends. The two adjacent MCs were exposed by a single longitudinal incision placed between them while in case of multiple MC fractures; the incision was given between the second and third or fourth and fifth MCs. The first MC was explored between extensor pollicis longus and extensor pollicis brevis. In the proximal phalanx, the fracture was exposed either through a dorsal vertical or a lazy S incision or mid-lateral incision which extended beyond neighboring joints. The incision over the extensor tendon in the middle phalanx was paramedian without violating the insertion of the central slip at the base of the middle phalanx. The PIP joint was exposed between the insertion of the extensor communis tendon and the interosseous tendon. The collateral ligament was cut and re-sutured at the end of the procedure using 4-0 catgut. Exposure of the distal interphalangeal (DIP) joint was performed through a dorsal approach through an H-shaped incision, which provided exposure to extensor aponeurosis and the distal part of the middle phalanx. Screws were used of 1.5 mm (1 mm drill) for phalanges and 2 mm (1.5 mm drill) for MCs. Generally, mini-straight plates of a minimum of four holes were used, while in the case of proximal or distal fractures, a T plate or L plate was used. The plate was applied on the dorsal surface of MCs or phalanges in transverse or short oblique fractures. Long oblique and spiral fractures were fixed by additional inter-fragmentary screws and plate osteosynthesis. Adequate soft tissue coverage and meticulous hemostasis were achieved in all cases to prevent post-op hematoma and hardware prominence.

Post-operative management and evaluation

A gentle compression bandage with limb elevation and intra-venous antibiotics were given for 12 hours followed by oral antibiotics for two days and anti-inflammatory medication for five days. Active movement was encouraged as early as possible while stitches were removed on the 10th-12th post-operative day. Patients were evaluated at six weeks, 12 weeks, and six months or until union was achieved. Wax bath/contrast baths were supplemented with physiotherapy when the range of movement (ROM) was slower than expected. Failure to unite fractures at six months was considered a procedure failure. In each follow-up, a clinical assessment was done using the American Society for Surgery of the Hand (ASSH), total active flexion (TAF), total active range of motion (TAM) score, and the Disabilities of the Arm, Shoulder, and Hand (quick DASH) score. The radiologic assessment was done with sequential roentgenograms at each visit. According to the ASSH report, the total active flexion of all the joints in a normal finger is 260° (normal 260°= MP 100°+ PIP 100°+ DIP 60°) and for the thumb, it is 130° (normal 130°= MP 50°+ IP 80°). The value of TAM is a subtraction of TAF and total extension deficit (TED).

A quick DASH score consists of 11 items to measure physical function and systems with any or multiple musculoskeletal disorders of the upper limb and the final score ranges between 0 (no disability) to 100 (the greatest possible disability).

Statistical analysis

Biostatistical analysis of data compiled with Prism Five software presented as categorical (percentage) and normally distributed variables (mean and standard deviation). Quantitative variables were compared with the paired Student t-test and considered significant if the p-value was less than 0.05 (p < 0.05). Repeated measures of ANOVA scores were used to measure changes in mean scores over varied time points.

## Results

The most common patterns observed and treated were transverse fractures of the MC shaft followed by oblique fractures of the base and shaft of MCs. All the patterns of fractures, their affected sites, and treatment are compiled in Table 1. 

**Table 1:** Details of fracture patterns, parts of hand fractured and their type of fixations with hardware. Abbreviations: N: number of items, %: Percentage, MC: Metacarpal, PP: Proximal phalanx, MP Jt.: Metacarpal-phalangeal joint, IP Jt.: Interphalangeal joint

Fracture Pattern (N)	Parts of Hand Fractured (N,%). [Proximal Phalanx (PP; 18, 31%) and Metacarpal (MC; 40, 68.96%)]		Cases, N (%)	Type of fixation (Hardware)
Transverse Fracture (21,36%)	MC Shaft (15,25.86%)	2^nd ^MC shaft	2 (3.45%)	Plate with 4 screws
		3^rd^ MC shaft	2 (3.45%)	Plate with 4 screws
		4^th^ MC shaft	3 (5.17%)	Plate with 4 screws
		4^th^ MC shaft	5 (8.62%)	Plate with 5 screws
		4^th^ MC shaft	3 (5.17%)	Plate with 6 screws
	PP Base (2, 3.45%)	5^th^ PP base	2 (3.45%)	L Plate with 4 screws
	PP Shaft (4, 6.9%)	3^rd ^PP shaft	1 (1.72%)	L plate with 4 screws
		4^th^ PP shaft	3 (5.17%)	Plate with 4 screws
Short Oblique Fracture (22, 37.9%)	MC Base (9, 15.5%)	1^st^ MC base	5 (8.62%)	T plate with 6 screws
		1^st^ MC base	1 (1.72%)	T plate with 5 screws and 1 inter-fragmentary screw
		4^th^ MC base	2 (3.45%)	L Plate with 4 screws
		5^th^ MC base	1 (1.72%)	T Plate with 4 screws and 1 inter-fragmentary screw
	MC Shaft (7, 12.06%)	2^nd^ MC shaft	2 (3.45%)	Plate with 6 screws
		3^rd^ MC shaft	2 (3.45%)	Plate with 4 screws
		5^th^ MC shaft	3 (5.17%)	Plate with 4 screws
	PP Base (3, 5.17%)	5^th^ PP base	3 (5.17%)	L plate with 4 screws
	PP Shaft (3, 5.17%)	3^rd^ PP shaft	3 (5.17%)	L plate with 4 screws
Long Oblique Fracture (11,18.96%)	MC Base (2, 3.45%)	3rd MC base	2 (3.45%)	T plate with 4 screws and 1 inter-fragmentary screw
	MC Shaft (6, 10.34%)	2^nd^ MC shaft	1 (1.72%	Plate with 4 screws and 1 inter-fragmentary screw
		4^th^ MC shaft	3(5.17%)	Plate with 5 screws and 1 inter-fragmentary screw
		5^th^ MC shaft	2 (3.45%)	Plate with 4 screws and 1 inter-fragmentary screw
	PP Base (1, 1.72%)	2^nd^ PP base	1 (1.72%)	L plate with 4 screws and 1 inter-fragmentary screw
	PP Shaft (2, 3.45%)	3^rd^ PP shaft	2 (3.45%)	Plate with 4 screws and 1 inter-fragmentary screw
Intra-articular Extension (4, 6.89%)	Metacarpal (1, 1.72%)	MC with MP Jt.	1 (1.72%)	L Plate with 2 screws
	Proximal Phalanx (3, 5.17%)	PP with MP Jt.	1 (1.72%)	L Plate with 2 screws
		PP with IP Jt.	2 (3.45%)	T Plate with 4 screws
		Total	58 (100%)	

The mean age of patients was 34.04 + 12.57 years (range, 19-60 years) and the mean follow-up was 14.28+ 2.85 months (range, 6-19 months). Most of the patients were in the age group of 21-50 years (38 cases;76%) followed by six (12%) cases each in the 18-20 and 51-60-year age groups. Most of the patients were confined to the male sex (41 (82%) male; nine (18%) females) with predominantly injuries over the right hand, about twice as much as their left hand. Occupations of the patients varied from skilled (n, 17 (34%)), semiskilled (n, 28 (56%)) to unskilled professions (n, 5 (10%)), and all of them complained of a significant loss to their work after an injury. The common modes of injuries were fall on hand (16; 32%), roadside accident (15; 30%), struck by a hard object (10; 20%) punching injury (7; 14%), and machine injury (2; 4%). Only eight (16%) subjects had fractures of two bones in the same hand but there was no involvement of bilateral hands in any case. Two cases had clavicle fractures, two had Colle’s fractures in contralateral hands and one patient had Galeazzi fracture in the ipsilateral limb. Five patients also had additional injuries, including Pott’s fracture in two (4%), metatarsal fracture in one (2%), proximal tibia fracture in one (2%), and medial malleolus fracture in one (2%). Modes of anesthesia for surgical intervention were wrist block, brachial block, ring block, and general anesthesia in 35 (70%), 10 (20%), two (4%), and three (6%) cases, respectively. All the fractures had perfect union clinically as well as radiologically at a mean duration of 6.24+1.08 weeks (range, 4-9 weeks). Mean TAM was 226.55+57.06, 228.79+54.11, and 231.03+52.29 at six weeks, 12 weeks, and six months, respectively, while contralateral fingers had a mean value of 241.89+47.46. 44 (88%) cases had achieved excellent results with a range of TAM being more than 75% of a contralateral finger. Gradation of TAM results using ASSH Score at six weeks, 12 weeks, and six months follow-up is described and compared with normal contralateral hand in Table 2.

**Table 2:** Gradation of TAM results using ASSH Score at six weeks, 12 weeks, and six months follow-up and compared with normal contralateral hand. Abbreviations: TAM: Total active motion, ASSH: American Society for Surgery of the Hand, Std. Deviation: Standard Deviation, %: percentage

TAM (in degrees)		Mean (degrees)	Median (degrees)	Std. Deviation (degrees)	Range (degrees)	P-values between different follow-ups (significance, p <0.05), Student t-test		
						6-12 weeks	12 weeks-6 months	6 weeks-6 months
Operated Hand	6 weeks	226.55	255	57.06	175	0.051	0.045	0.122
	12 weeks	228.79	255	54.11	170			
	6 months	231.03	255	52.29	170			
Non-operated Hand finger		241.89	260	47.46	150			
TAM (in percentage)		Mean (%)	Median (%)	Std. Deviation (%)	Range (%)	P-values between different follow-ups (significance, p < 0.05), Student t-test		
						6-12 weeks	12 weeks-6 months	6 weeks-6 months
Operated Hand	6 weeks	93.49	100	13.19	46.29	0.0386	0.0461	0.104
	12 weeks	94.44	100	10.92	37.04			
	6 months	95.32	100	9.04	31.48			
Non-operated Hand		100	100	0	0			

The mean pre-operative quick DASH score was 72.2+2.53 and improved to 43.7+10.61, 4.4+9.09, and 2+4.95 at six weeks, 12 weeks, and six months, respectively. Forty-six (92%) hands had an excellent functional outcome whereas only four (8%) had good functional outcomes as per quick DASH score. The differences over the period of time between follow-ups to assess quick DASH scores were statistically significant (p < 0.001) and compiled in Table 3.

**Table 3:** Post-op functional outcomes of operated finger/hand using quick DASH (Disability of Arm, Shoulder and Hand) score at six weeks, 12 weeks, and six months follow-up. Abbreviations: DASH: The Disabilities of the arm, shoulder and hand, ANOVA: Analysis of variance, NA: Not applicable

Quick DASH Score	Mean	Median	Std. Deviation	Range	P-value (Student t-test for difference from pre-op time), (Repeated measures of ANOVA for difference in groups over the duration), (Significance p<0.05)			
					(Student t-test)	(Repeated measures of ANOVA for difference in groups over the duration)		
						6 weeks-12 weeks.	12 weeks-6months	6weeks-6months
Pre-operative	72.2	70	2.53	5	NA			
At 6 weeks	43.7	41.25	10.61	37.5	<0.01	<0.01	<0.01	<0.01
At 12 weeks	4.4	0	9.09	32.5	<0.01			
At 6 months	2	0	4.95	20	<0.01			

**Figure 1:**
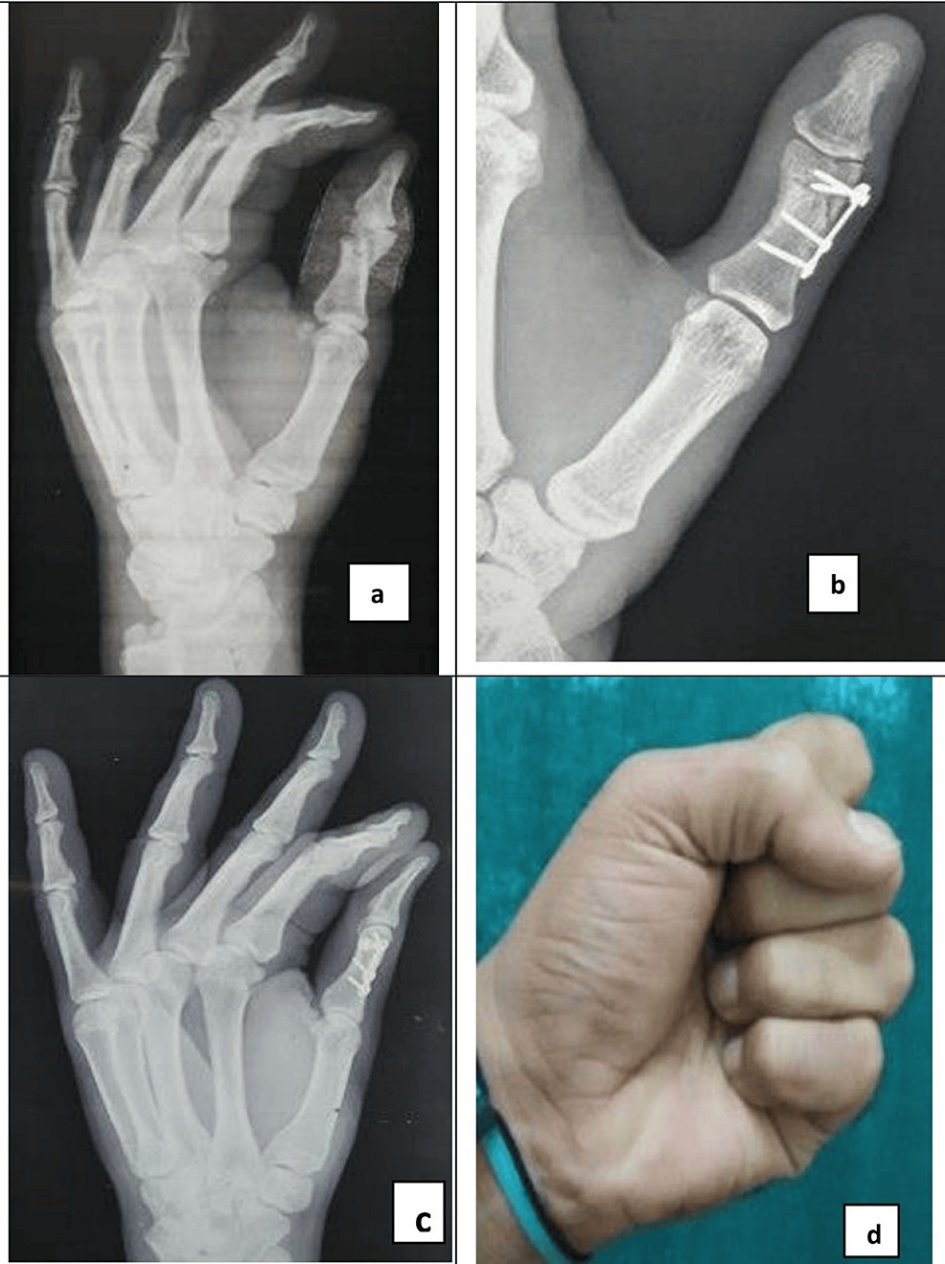
Radiograph showing Intra-articular fracture of 1st proximal phalanx (a) and after open reduction and fixation with a T plate (b). Radiograph showing complete union at fracture site (c), and clinical picture showing excellent active range of motion (d).

**Figure 2:**
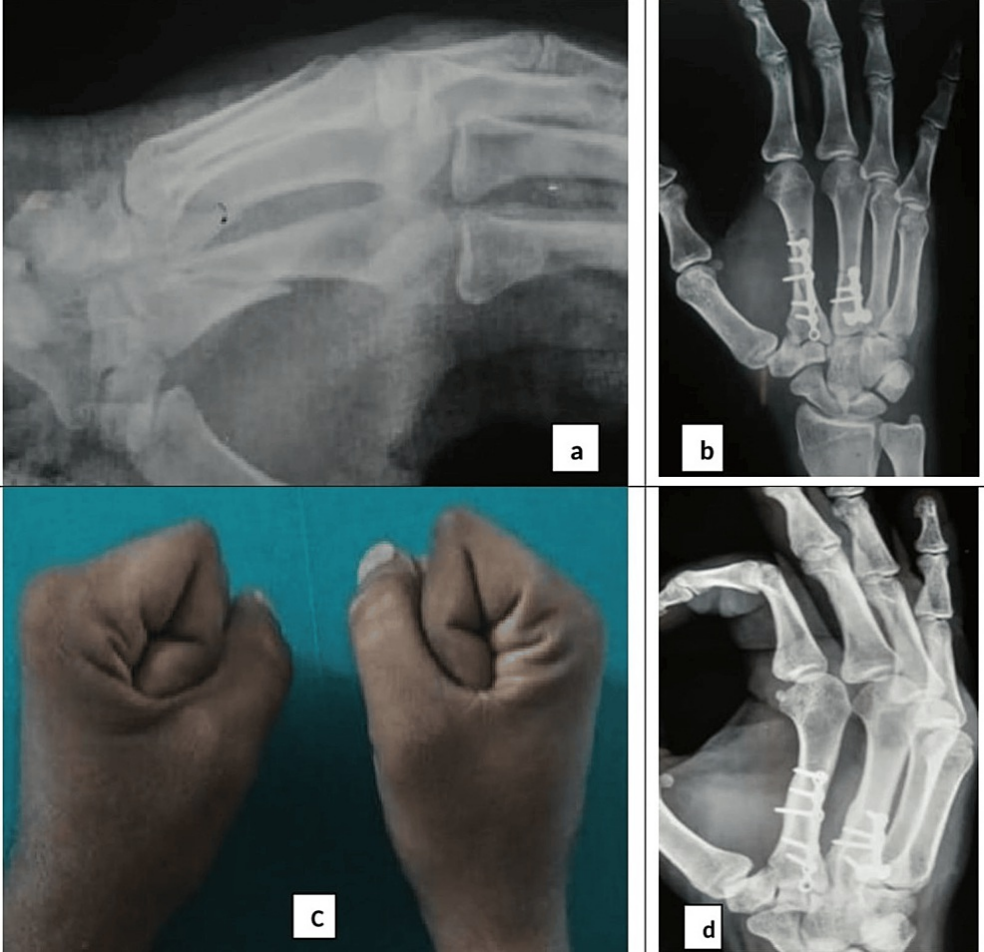
Radiographs showing oblique fracture of 2nd and 3rd Metacarpals (a), ORIF with a plate and interfragmentary screws (b), clinical picture showing comparable excellent active ROM (c), radiograph showing complete union at fracture sites (d).

**Figure 3:**
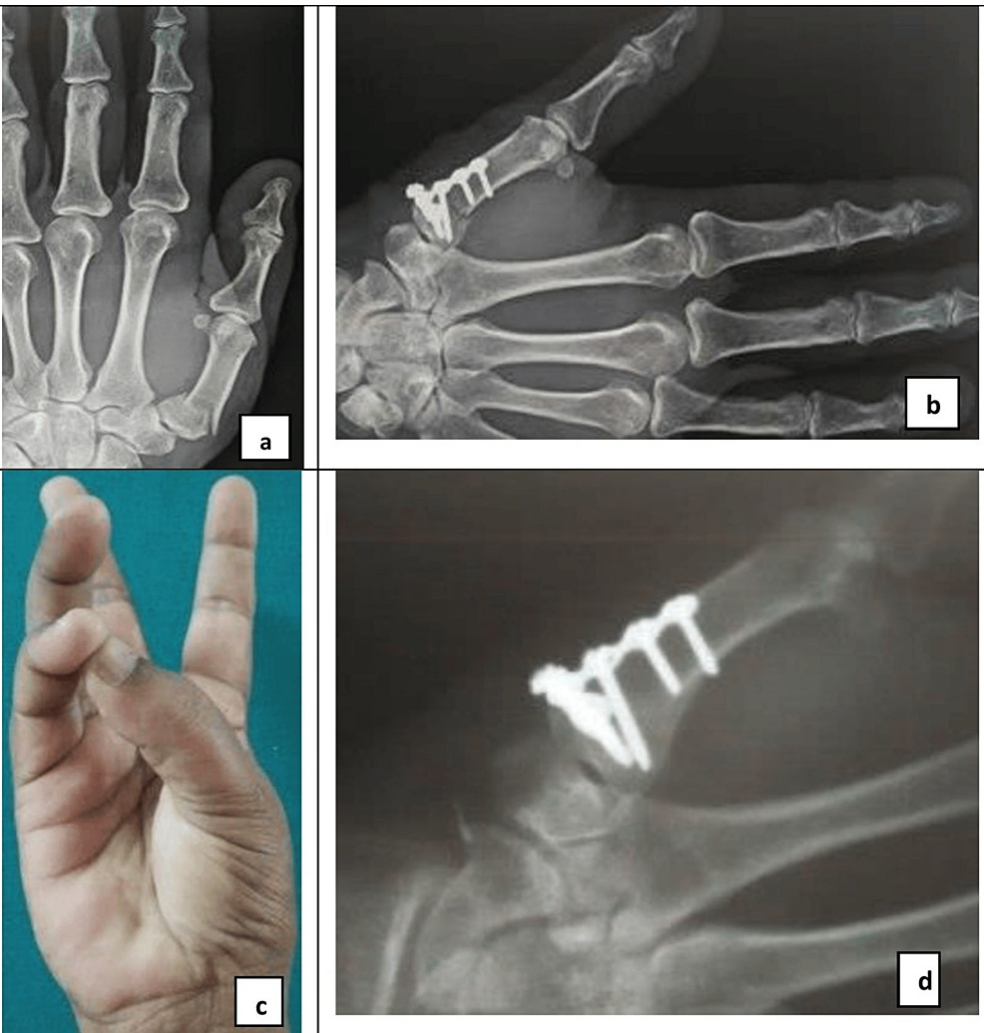
Radiographs showing Oblique fracture of 1st Metacarpal (a), open reduction/Fixation with a T plate (b). Clinical picture showing excellent active range of motion and (c), radiograph showing complete union at fracture site (d).

All MC fractures had excellent ASSH functional outcomes while phalangeal fractures had excellent and fair outcomes in 70% (13) and 30% (5) cases. Similarly, 100% MC fractures had excellent quick DASH functional outcomes while for phalangeal fractures outcomes were excellent and good in 77.78% and 22.22 % cases. Pre-operative fracture patterns, their management with different implants (plates and screws), and clinical and functional outcomes at the final follow-up of first proximal phalanx, second /third MCs, and base of first MC fractures are illustrated in Figures 1-3, respectively.

No one developed skin necrosis, permanent edema, non-union, or osteomyelitis. Stiffness and extensor tendon adhesions were encountered in seven cases. They gained considerable improvement after aggressive physiotherapy with contrast baths and were able to indulge productively in their routine activities. Three cases developed superficial stitch infections and were managed with aseptic dressing, removal of stitches, and antibiotics. Two patients with a short oblique fracture of the fifth PP base complained of mild pain during a three-month follow-up which was relieved completely by medication and contrast baths. Two cases of transverse fracture of the PP shaft developed malunion that restricted total active ROM, but functional ROM improved with active physiotherapy. There was no event of any loss of reduction, implant breakage, loosening, or tendon irritation at any operative site. Complications observed after surgical intervention (ORIF with plating) are compiled in Table 4.

**Table 4:** Post-operative complication observed after open reduction and internal fixation with miniplates and screws. Abbreviations: %: Percentage

SN	Complications	N (% of fractures)
1	Stiffness	7 (12.06%)
2	Superficial infection	3 (5.17%)
3	Edema	1 (1.72%)
4	Pain	3 (5.17%)
5	Mal-union	2 (3.44%)
6	Non-union	0 (0%)
7	Collateral ligament instability	0 (0%)
8	Loss of reduction	0 (0%)
9	Loosening of implant	0 (0%)
10	Skin necrosis	0 (0%)
11	Osteomyelitis	0 (0%)

## Discussion

Hands are engaged in fine and skilled activities so functional outcomes are more appreciable than anatomical healing as compared to other bones of extremities. Stark mentioned that excellent results could be achieved in selective fractures with improved surgical tools and techniques, better anesthesia, the use of magnification, and modern surgical skills [6]. In displaced fractures, rotatory deformities or overriding can lead to finger malalignments, tendon adhesions, and decreased power to grasp in the majority, when managed conservatively with a non-anatomical reduction [6]. Overall, successful outcomes of these fractures require a clear appreciation of fractured anatomy and patterns. The need for ORIF in these fractures is documented very sparsely in past literature, consisted around 5% of all [10,11].

The study incorporated only closed fractures and low-grade compound fractures to subside hampered functional outcomes associated with soft tissue injuries. According to Huffaker et al., the associated soft tissue insult in the form of crush injury, tendon injury, or skin affection reduced the final ROM of both fractured and unfractured fingers of the same hand [10]. Some well-documented studies concluded better functional outcomes in closed fracture fixation than compound one in terms of total ROM [10-15]. Male (82%) preponderance was noticed, similar to other past studies by Crawford et al. (90%), Jupiter et al. (95%), and Basar et al. (83%) [11-13]. This might be due to trauma associated with more involvement of males by the nature of their outdoor activities and profession especially in developing countries. Fixation of transverse fractures of MCs or shafts of phalanges with k-wires is a traditional option but fixation may not be so rigid to provide rotational stability [8,13].

External fixation for compound and closed fractures has not been popular because of cumbersomeness and high morbidity [7,10,15]. There is no joint transfixation in minifixators thus avoiding stiffness of nearer joints, resulting in better outcomes. Elastic intramedullary small nails or wires can be used for oblique and transverse fractures of diaphyseal fractures of the hand [7,8,15-18]. Ozer et al. compared the fixation of the extra-articular MC fracture with these intramedullary wires and plate-screws, which concluded no significant difference in the functional outcome but better compliance and less complications in the plate-screws group [18]. Lag-screw fixation may be the best choice for open fixation of long oblique phalangeal and MC fractures, while for short oblique fractures, plating or tension band wiring is recommended [19]. Although plate fixation for MCs is advocated by almost all authors in phalanges, it is a subject of controversy because of tendon problems and stiffness [19,20]. Basar et al. concluded that miniplate osteosynthesis for unstable MC fractures allowed early mobilization by providing rigid anatomical reduction, thereby preventing stiffness and hence good functional results [13]. Soni et al. observed 100% union rates and excellent functional outcomes in 85.71% of patients, where closed ipsilateral multiple MC fractures were treated with miniature plate fixation, which was concluded viable and excellent option for treatment [21].

We performed ORIF with plates and inter-fragmentary screws in 11 long oblique and two short oblique fracture patterns (10 MC, 3 PP) and have been well advocated by several studies [11,20,22-24]. Crawford observed excellent outcomes of screw fixation for proximal phalangeal fractures, while for MC fractures outcomes were similar to conservative means [11]. This is probably due to more rotational instabilities in long oblique MC fractures than similar phalangeal fractures. The present study overcame this instability by inserting an inter-fragmentary screw and strengthening it with a plate. Drilling for inserting an inter-fragmentary screw was a difficult observation in our study, as the smaller size of bone does not allow any latitude for error in drilling and is also supported by Meyer et al. and Melone et al. [23,24].

Radiological and functional outcomes

Moreover, it was observed, and also literature revealed that excellent radiologic results are not always accompanied by an excellent function [24]. All fractures had perfect union and were probably explained by adequate fixation and high vascularity of the small bones of the hand. Similarly, Omokawa et al. reported 100 % bone union in all 51 fractures over an average period of 2.6 months [25]. Our results are comparable to studies by Gupta et al., Soni et al., and Trevisan et al. [14,21,26].

We obtained excellent results (TAM >220°) in 45 cases (90%), while five (10% cases had fair results (TAM 180°-220°). Gupta et al. found TAM >220° in all patients in the group where fractures were fixed with plate screws [14]. Souer et al. evaluated the results of plate fixation in a series of closed ipsilateral multiple MC fractures and found a TAM > 230° in 18 of 19 (94.73%) patients [27]. Soni et al. observed an excellent function outcome in 18 of 21 (85.71%) patients with closed multiple MC fractures [21]. Trevisan et al. observed excellent TAM in 41 out of 44 (93.18%) patients [26]. Similarly, Başar et al., Tan et al., Bosscha et al., and Joshi et al. observed excellent TAM in 88.37%, 79%, 92%, and 87.5% of patients, respectively [13,28-30].

Mean TAM was observed 226.55°+57.06° at six weeks, which improved to 228.79°+ 54.11° at 12 weeks and further to 231.03°+52.29° at six months. Trevisan et al. observed a mean TAM of 256° (range 175°- 260°), while Joshi et al. found a mean TAM of 261.76+24.87 in the final follow-up [26,30]. Mean TAM% improved from 93.49+13.19 at six weeks to 94.44+10.92 and 95.32+9.04 at 12 weeks and six months, respectively. Two out of five cases whose outcomes were fair in early follow-up improved to excellent at 12 weeks while the remaining three cases finally had a fair TAM score at the end of six months.

Rigid miniature plate and screw fixation by allowing early movements reduced the period of rehabilitation and work loss reflected by excellent quick DASH scores. 46 (92%) patients had excellent and four (8%) had good functional outcomes in terms of quick DASH scores. The mean quick DASH score of 72.2+2.53 was improved to 43.7+10.61 (range 30-67.5) and 4.4+9.09 (range 0-32.5) at six weeks and 12 weeks, respectively, which further improved to 2+4.94 (range 0-20) at six months follow up. The difference over the period of time was statistically significant with a value of <0.001. Soni et al., in their series of 21 patients, observed a mean quick DASH score of 8.47 (range 1-26) [21].

The results between MCs and phalanges were comparable and had a mean TAM of 236.84°+54.42° and 220°+48.76°, respectively. A mean quick DASH score of 0.47+1.87 with excellent results was found in all MC fractures; whereas in phalangeal fractures, it was 4.72+7.55 with excellent results in 14 out of 18 (78%) patients. Excellent TAM scores were gained for all MC fractures (40) as compared to 14 out of 18 (78%) phalangeal fractures. These results (<220°) were related to a short oblique fracture of the fifth PP base and could be of the small size of the proximal phalanx, leading to tendon adhesions and stiffness. Gupta et al. observed excellent and good functional outcomes in 94% of MC fractures and 54% of phalangeal fractures [14]. Similarly, Ruedi et al. reviewed 100 fractures of hand treated by ORIF (ASIF mini set) and resulted in better functional results in MC fractures than finger fractures [22].

The strengths of this case series are good sample size, all patients being managed by one senior orthopedic surgeon in a dedicated center, and good follow-up. The main limitation is that there is no comparison group. A prospective study with one large cohort of patients or a multicenter study with a comparable group will help overcome the limitations of this study.

## Conclusions

We conclude that plate fixation provides enough stability in metacarpals and phalanges fractures, thereby allowing immediate active movements, which leads to excellent functional outcomes and early return to normal activities. Inter-fragmentary screws have a definitive role in long oblique fractures for excellent functional results. Fractures of the proximal phalanx shaft gave excellent functional results compared to base and intra-articular fractures involving DIP and IP joints.
